# The “Social Gaze Space”: A Taxonomy for Gaze-Based Communication in Triadic Interactions

**DOI:** 10.3389/fpsyg.2018.00226

**Published:** 2018-02-26

**Authors:** Mathis Jording, Arne Hartz, Gary Bente, Martin Schulte-Rüther, Kai Vogeley

**Affiliations:** ^1^Department of Psychiatry and Psychotherapy, University Hospital Cologne, Cologne, Germany; ^2^JARA-BRAIN, Aachen, Germany; ^3^Translational Brain Research in Psychiatry and Neurology, Department of Child and Adolescent Psychiatry, Psychosomatics, and Psychotherapy, University Hospital RWTH Aachen, Aachen, Germany; ^4^Department of Communication, Michigan State University, East Lansing, MI, United States; ^5^Cognitive Neuroscience (INM-3), Institute of Neuroscience and Medicine, Research Center Jülich, Jülich, Germany

**Keywords:** non-verbal communication, social gaze, joint attention, triadic interaction, ecological validity, taxonomy, social psychology

## Abstract

Humans substantially rely on non-verbal cues in their communication and interaction with others. The eyes represent a “simultaneous input-output device”: While we observe others and obtain information about their mental states (including feelings, thoughts, and intentions-to-act), our gaze simultaneously provides information about our own attention and inner experiences. This substantiates its pivotal role for the coordination of communication. The communicative and coordinative capacities – and their phylogenetic and ontogenetic impacts – become fully apparent in triadic interactions constituted in its simplest form by two persons and an object. Technological advances have sparked renewed interest in social gaze and provide new methodological approaches. Here we introduce the ‘Social Gaze Space’ as a new conceptual framework for the systematic study of gaze behavior during social information processing. It covers all possible categorical states, namely ‘partner-oriented,’ ‘object-oriented,’ ‘introspective,’ ‘initiating joint attention,’ and ‘responding joint attention.’ Different combinations of these states explain several interpersonal phenomena. We argue that this taxonomy distinguishes the most relevant interactional states along their distinctive features, and will showcase the implications for prominent social gaze phenomena. The taxonomy allows to identify research desiderates that have been neglected so far. We argue for a systematic investigation of these phenomena and discuss some related methodological issues.

## Social Gaze as Special Case of Non-verbal Communication

Non-verbal communication does not only supplement verbal utterances but constitutes a crucial part of communication in itself. Thereby, non-verbal communication must not be treated as a series of isolated and discrete signals but as a complex and dynamic process (Burgoon et al., 1989, p. 23). In addition, the production and perception of non-verbal communication behavior are often implicit and automatic (Choi et al., 2005) – i.e., unintentional, uncontrollable processes humans are unaware of (Bargh, 1994).

Among the non-verbal cues, gaze behavior plays a pivotal role. The eyes are among the first and most frequently fixated regions in humans (Yarbus, 1967; Walker-Smith et al., 1977) from early infancy on (Haith et al., 1977), serve face and emotion recognition, and allow to identify gender, age, and personality (George and Conty, 2008; Itier and Batty, 2009).

The morphology of the human eye with its white sclera significantly enhances the visibility of the eyes and facilitates gaze recognition (Kobayashi and Kohshima, 1997, 2001), suggesting evolutionary adaptation to the increased importance of gaze-based social interaction and, eventually, social cognition in humans (Emery, 2000). Ontogenetically, attending to gaze can be considered a precursor of cooperation in young children (Tomasello et al., 2007). Both phylogenetically and ontogenetically (Grossmann, 2017) social gaze opens a “window into social cognition” (Shepherd, 2010).

In addition to coordination and management of verbal conversation (Argyle and Cook, 1976), gaze mutually coordinates attention which is a hallmark of social learning, communication, social interaction, and, finally, shared intentionality (Tomasello et al., 2007) and joint action (Sebanz and Knoblich, 2009). So-called joint attention (JA) is typically defined in the gaze domain: In triadic interactions (e.g., Lee et al., 1998), two persons can jointly attend to an object by one person following another person’s gaze toward a given object or possibly a third person. JA is the basis and prerequisite of cooperation (Tomasello et al., 2007) and has been investigated in great detail (Kleinke, 1986; Emery, 2000; Frischen et al., 2007; George and Conty, 2008; Itier and Batty, 2009; Shepherd, 2010; Falck-Ytter and von Hofsten, 2011; Pfeiffer et al., 2013b; Oberwelland et al., 2016; Grossmann, 2017).

## The “Social Gaze Space” (SGS)

Despite the wealth of social gaze research, a unifying taxonomy of social gaze is still lacking. For the most commonly used taxonomy Emery (2000) summarized several core processes like averted gaze, mutual gaze, gaze following and JA under the term social gaze. However, this taxonomy has two major limitations: (1) the basic processes described by Emery were not considered as extended in time. Relatedly, transitions between states have not been taken into account. The taxonomy of Emery therefore lacks the complex and dynamic character of gaze encounters between two persons, which are extended in time and are based on the continuous exchange between the interactants. (2) An additional restriction of the traditional social gaze terminology and research is that they focus on explicit interactions in which at least one person deliberately tries to interact with or respond to another (Schilbach et al., 2010; Pfeiffer et al., 2014). However, already the mere presence of another person presumably strongly affects a persons’ behavior even when the partner is not interactively engaged. Recent research about the dual function of social gaze demonstrates that the awareness of someone else watching oneself can change the own gaze behavior (Gobel et al., 2015; Jarick and Kingstone, 2015). In accordance with recent interactionist advances emphasizing the dynamical character of interactions and arguing for ecological validity (Risko et al., 2012, 2016; Pfeiffer et al., 2013a; Schilbach et al., 2013), it is therefore important to consider all possible states of triadic interactions in a holistic approach.

**FIGURE 1:**
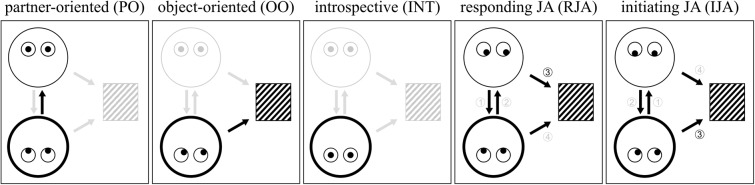
Illustration of the five interactional states of the SGS (illustration in alignment with Emery, 2000) from the perspective of index person A (always the bold face at the bottom) in interaction with person B. (1) Partner-oriented: Attentional focus of A is directed toward B without deliberate attempts to interact of any of the two interactants. (2) Object-oriented: Attentional focus of A is directed toward an object within the shared environment. (3) Introspective: The attention is directed toward A’s own inner experience. (4) Responding JA: A follows B’s gaze toward an object. (5) Initiating JA: A tries to shift B’s attention toward an object.

**FIGURE 2:**
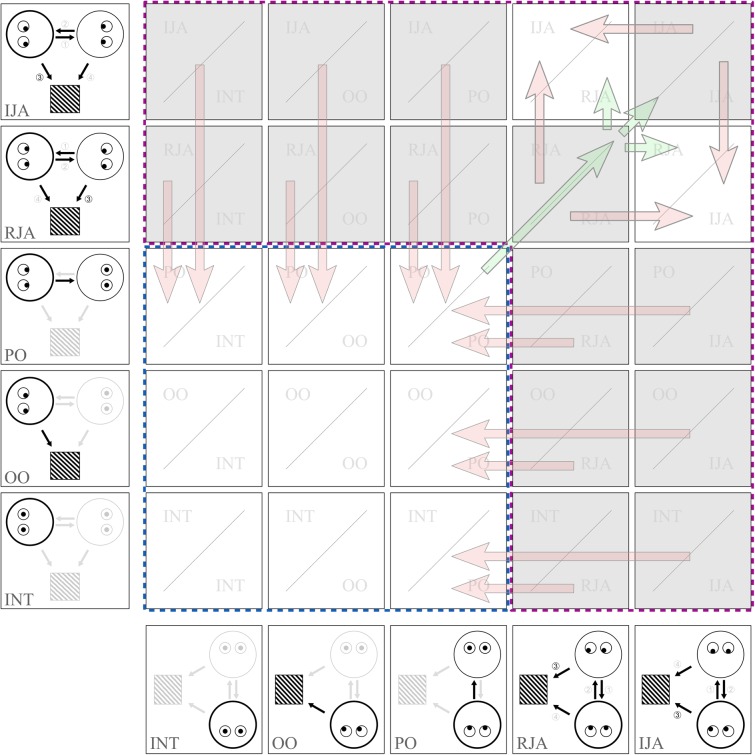
All possible dual gaze states as a result of the combinations of gaze states of the two interactants (*x*- and *y*-axis). For illustration purposes states are presented in different order than previously introduced and as compared to **Figure 1**. Cell color indicates compatibility and stability of the states with white denoting compatible/stable states and gray denoting incompatible/unstable states. Red arrows suggest transitions from unstable to stable states. Green arrows exemplify the establishment of an interaction with a state of mutual interest serving as origin or gate (Note that arrows are not exhaustive of all possible transitions). The blue box (blue dashed line) designates states which methodologically can be inferred from a separate analysis of each participant. The purple box (purple dashed line) designates states which can only be inferred by an analysis of dynamics and interdependencies between the interactants.

In the following, we propose a taxonomy of the “Social Gaze Space” (SGS) that comprises all internal states a person can possibly adopt in the most basic setup of a gaze-based triadic interaction, as constituted by two interaction partners and an object^1^. These states are: partner-oriented (PO), object-oriented (OO), introspective (INT), responding joint attention (RJA), and initiating joint attention (IJA). We define these states on the basis of the behavior of one interactant (**Figure 1**). A dynamic interaction involving two persons can be conceptualized as a combination of two out of five different states which need not necessarily be temporally aligned. All combinations of states are possible and generate different types of interactional encounters that can be represented as a two-dimensional series of social gaze states evolving in time (**Figure 2**). This particularly applies to the interactive states of RJA and IJA, in which a person attempts to engage another person in an interaction which can be successful or not (see below section Triadic Interaction as a Dynamic Function of a Two-Dimensional State-Space). For this conceptualization, our focus lies on overt visual attention as deducible from gaze direction, whereas covert attention and other correlates of attention (e.g., pupil diameter, eye convergence, blinking rate) will be discussed only marginally.

## The Five Gaze States

### Partner-Oriented (PO)

In the partner-oriented state person A focuses her attention on person B. The eyes automatically attract visual attention (Laidlaw et al., 2012) and possibly convey information about personal attributes including gender, age and identity (Schyns et al., 2002), as well as emotional and attentional states (Baron-Cohen et al., 1997; Emery, 2000).

Eyes that focus on the viewer will be preferentially looked at (Senju and Hasegawa, 2005) or evaluated much more positively (Stass and Willis, 1967), modulate attention (Senju and Hasegawa, 2005; Dalmaso et al., 2017), increase emotional empathy (Schulte-Rüther et al., 2007) and modulate cognition suggesting a substantial ‘eye-contact-effect’ for diverse aspects of socio-emotional perception (Senju and Johnson, 2009). Among distractor stimuli, viewer-directed gaze is detected easily and much faster than averted gaze (von Grünau and Anston, 1995; Conty et al., 2006; Senju et al., 2008). Profound effects of viewer-direct eye gaze on preference (Hains and Muir, 1996) and attentional modulation (Farroni et al., 2002) have also been demonstrated in infants. This is probably the most thoroughly studied gaze state.

The effect of eye contact is much stronger during dynamic interactions with real persons than when confronted with static pictures (Hietanen et al., 2008; Pönkänen et al., 2011). This requires interactive approaches with dynamic face-to-face interactions (Pfeiffer et al., 2012, 2013a; Risko et al., 2012, 2016; Schilbach et al., 2013; Schilbach, 2014; Oberwelland et al., 2016).

### Object-Oriented (OO)

In the object-oriented state person A’s attention is focused more or less entirely on an object in the shared environment, but not on the other person (as opposed to joint attention states described below during which person A oscillates between objects and person B). That is B’s presence and behavior are likely to influence A to some level but merely coincidentally and probably without A’s awareness. The exploration of different objects in a visual scenery is affected by the saliency of objects and thus the probability of persons directing their attention toward the objects (Itti and Koch, 2000). However, top-down as well as bottom-up processes are actively working together or compete for attention (Egeth and Yantis, 1997). Again, our attention and behavior toward objects are altered by actions or even the mere presence of another person looking at us (Senju and Hasegawa, 2005). Gaze cueing can automatically lead the attention toward particular objects (Frischen et al., 2007), even overriding the effect of higher psychophysical saliency (Borji et al., 2014). This brief instance of social interaction might induce a lasting attentional shift from a state of OO to the state of RJA [as examined in section Responding Joint Attention (RJA)]. However, even in the absence of any active gaze cuing, the presence of another person can attract covert attention (Kuhn et al., 2016; Laidlaw et al., 2016). Furthermore, the mere knowledge of the possibility of someone else watching their gaze lets participants control their gaze behavior with respect to its social adequacy (Risko and Kingstone, 2011).

### Introspective (INT)

In this state person A neither focuses on objects nor on persons in the environment but only on his inner experience. Attentional disengagement from the outside world has been shown to correlate with a decrease in saccade frequency and an increase in saccade amplitude (Benedek et al., 2017) and, accordingly, a decrease in fixation frequency and an increase in fixation duration (Reichle et al., 2010; Benedek et al., 2017). Furthermore, in these situations blinking rate can increase (Smilek et al., 2010) and blinking duration can be prolonged (Salvi et al., 2015; Benedek et al., 2017). INT seems to show more variability in pupil diameter than episodes of directed attention to outward stimuli (Smallwood et al., 2011; Benedek et al., 2017). A higher variability of eye vergence (Benedek et al., 2017) suggests a less focused gaze (Solé Puig et al., 2013).

While it is intuitively obvious that these changes are indicative of a reduced responsiveness to events in the outside world (Smallwood et al., 2011; Benedek et al., 2017), it is an open question whether the reduced responsiveness to external stimuli and the overall change in gaze behavior are both the result and an epiphenomenon of INT, or whether changes such as a decrease in the frequency of microsaccades during INT may represent active visual disengagement as a strategy to achieve reduced responsiveness (Benedek et al., 2017). Another strategy participants adopt in situations of high cognitive load is to avoid looking into the eyes of an observer because this would entail higher demands on cognitive processing (Glenberg et al., 1998; Doherty-Sneddon and Phelps, 2005; Phelps et al., 2006; Markson and Paterson, 2009). Interestingly, the additional cognitive demands of mutual gaze do not seem to originate in the physical properties of the stimulus (e.g., the eyes) but in the interactive character inherent in this situation (Markson and Paterson, 2009). It is therefore crucial to consider introspective attentional states as potentially socially influenced by the presence of another person.

### Responding Joint Attention (RJA)

In the responding JA state person A waits for B to initiate and lead the interaction, e.g., B chooses an object and A follows B’s gaze toward the object. Gaze following reactions that respond to the invitation of another person thereby establishing a rudimentary form of JA appear to be deeply rooted in human behavior (Pfeiffer et al., 2011). The gaze of another person automatically cues one’s own attention even when it is uninformative (Friesen and Kingstone, 1998), and participants exhibit gaze following even for forthright counter-predictive gaze cues (Driver et al., 1999; Bayliss and Tipper, 2006).

Gaze following with the aim of establishing JA constitutes a very simple though effective mechanism allowing for the inference of the attentional focus of other persons. The ability to adopt the attentional focus of another person is a prerequisite for reinforcement learning, from infants to adults (Vernetti et al., 2017). Infants at 6 months of age are already able to follow the eyes of other persons, in particular in a communicative context (Senju and Csibra, 2008). Accordingly, early proficiency in gaze following in infants predicts the development of mentalizing and emergence of language (Morales et al., 1998; Charman et al., 2000). JA and gaze following facilitate social learning, social competence, self-regulation, intelligence, and depth of information processing (Mundy and Newell, 2007).

### Initiating Joint Attention (IJA)

In this state, person A takes the lead within the interaction by initiating JA. While gaze following in RJA reflects person A’s understanding that B’s perception and actions are goal-directed or have communicative intent, the initiation of JA is considered to require elaborate processing and insight (Tomasello and Carpenter, 2005). To initiate JA, A has to acknowledge (1) the dual function of social gaze (Gobel et al., 2015; Jarick and Kingstone, 2015) i.e., that gaze does not only serves her in perceiving but also that her gaze informs B about her focus of attention and, (2) sharing of attention is a desirable aim for mutual interaction (Tomasello et al., 2005). Whereas first elements of RJA are already evident at 6 months of age, IJA does not emerge before the second year of life (Mundy and Newell, 2007; Mundy et al., 2007). Chimpanzees followed the experimenters gaze on a frequent basis but did not try to initiate JA (Tomasello and Carpenter, 2005). Interestingly, differential development of both RJA and IJA can be observed in brain systems from childhood to adulthood (Oberwelland et al., 2016), as well as during atypical development in disorders such as autism (Oberwelland et al., 2017). In autism, IJA is typically more impaired than RJA and emerges much later than in typical development (Mundy, 2003). These empirical findings clearly point toward separate underlying cognitive systems of RJA and IJA (Mundy and Newell, 2007).

The innate tendency to expect other humans to follow their gaze (Pfeiffer et al., 2011) corresponds to the perception of successful initiation of JA as rewarding (Schilbach et al., 2010; Pfeiffer et al., 2014; Oberwelland et al., 2016). A successfully initiated instance of JA alters the consecutive interaction by increasing the tendency to look at and dwell upon the partners face (Bayliss et al., 2013).

### Triadic Interaction as a Dynamic Function of a Two-Dimensional State-Space

Having defined the basic states during triadic JA, the picture becomes more complex when considering that each of the two participants can adhere to any of these states during a triadic interaction unfolding in time. In theory, a dual social state may be one of 25 possible combinations (representing varying degrees of “interactivity”), spanning a two dimensional SGS (**Figure 2**; see McCall and Singer, 2015 for an alternative concept of a 2D gaze space). Some of these combinations might be more ephemeral than others: e.g., a person A might soon lose the motivation to initiate JA if person B does not respond to him adequately, person A might switch to PO very soon subsequently (‘stability’ of states is indicated by cell color in **Figure 2**, with gray cells indicating unstable and ephemeral states; red arrows represent subsequent shifts from unstable to stable states).

Furthermore, it is conceivable that mutual attention (PO/PO) might facilitate transitions from non-interactive to interactive states (indicated by green arrows in **Figure 2**). These transitions have yet to be empirically investigated. Only non-interactive states (blue box in **Figure 2**) can be understood on the basis of single persons whereas the study of interactive situations (purple box in **Figure 2**) requires a complex dynamic concept and experimental setup, based on the idea that the basic unit of analysis is the interaction between both interactants.

## Reflections and Future Directions

It is our goal to provide a unifying taxonomy of social gaze in triadic interactions and their respective interdependencies. This complex, dynamic and holistic approach has two major achievements. First, it facilitates the integration of existing empirical findings within one unifying framework and helps to identify research desiderates. Second, it will go beyond many of the previous studies that investigated gaze behavior in isolation and it will provide a theoretical background to study the complex dynamics of dual states including their transitions, thereby increasing the ecological validity of the empirical approaches.

This approach is in accordance with a growing number of proposals that argued in favor of “embedded” interactionist or “enactive” approaches and emphasize the importance of ecological validity in non-verbal communication and social cognition research (Kingstone, 2009; Marsh et al., 2009; De Jaegher et al., 2010; Konvalinka and Roepstorff, 2012; Risko et al., 2012, 2016; Skarratt et al., 2012; Gallagher, 2013; Pfeiffer et al., 2013b; Schilbach et al., 2013). New methodological approaches due to technological advances increasingly allow for the development of paradigms which meet those demands (Pfeiffer et al., 2013b; Oberwelland et al., 2016, 2017).

This paves the way to research questions concerning the nature of gaze communication in triadic interactions. Even in triadic encounters which are not explicitly interactive interactants are still likely to exert subtle influences on each other in many reciprocal ways: In PO, dynamic interactions elicit a much stronger eye contact effect that static pictures (Hietanen et al., 2008; Pönkänen et al., 2011); In OO, the visual attention of another person will influence object processing in an observer in multiple ways (Reid et al., 2004; Bayliss et al., 2006; Becchio et al., 2008); the oculomotor changes observable in INT might be an active form of visual disengagement (Benedek et al., 2017). Therefore, a separate examination of allegedly interactive and non-interactive states in triadic interactions is not adequate. From the new unifying perspective of the SGS the very first step must be to systematically describe and identify the characteristics of gaze behavior associated with the individual gaze states. However, given the dynamic and continuous nature of non-verbal communication (Burgoon et al., 1989) our appreciation of the interactants experience of the encounter relies on our comprehension of transitions between interactional states. The consequential next step will then be the identification of potentially complex signifiers of these transitions in gaze behavior, yet unknown (e.g., gaze patterns characteristic for active attempts to catch the partners attention to reach a full-fledged state of JA), which can serve as indicators of these transitions in future studies.

We speculate that transitions between gaze states of the individual interactants are not independent, but are contingent upon each other to a changing degree. If these contingencies are crucial in the establishment of states of higher interactivity and phenomena like synchrony and rapport between interactants, then it should be possible to establish their causal role in experimental paradigms. The dual state of mutual attention (PO/PO) as a candidate state for a gate to higher degrees of interactivity (**Figure 2**) – as soon as its role is empirically corroborated – could be a potential starting point in these investigations.

Having established the prototypical SGS it is worth studying individual differences in the behavior and experiences in triadic gaze interactions. Questions which to the best of our knowledge have not been tackled before concern the relationship between specific personality traits and gaze behavior in triadic encounters and to which degree personality traits are ascribed on the basis of gaze behavior. Other obvious topics relate to developmental factors in the SGS and how and when children access the SGS or the effect of impairments in non-verbal communication as observable in autism have in the SGS.

## Author Contributions

All authors substantially contributed to the conception of the work. MJ drafted the manuscript. AH, GB, MS-R, and KV revised it critically.

## Conflict of Interest Statement

The authors declare that the research was conducted in the absence of any commercial or financial relationships that could be construed as a potential conflict of interest.

## Footnotes

1 Although, our taxonomy explicitly comprises a set of discrete states, we use the term “Social Gaze Space” throughout the manuscript instead of the more precise term “Social Gaze State Space,” for the sake of simplicity and comprehensibility.
